# Oxidation of 2-Hydroxynevirapine, a Phenolic Metabolite of the Anti-HIV Drug Nevirapine: Evidence for an Unusual Pyridine Ring Contraction 

**DOI:** 10.3390/molecules17032616

**Published:** 2012-03-05

**Authors:** Alexandra M. M. Antunes, Muna Sidarus, David A. Novais, Shrika G. Harjivan, Pedro P. Santos, João L. Ferreira da Silva, Frederick A. Beland, M. Matilde Marques

**Affiliations:** 1 Centro de Química Estrutural, Instituto Superior Técnico, Universidade Técnica de Lisboa, Lisboa 1049-001, Portugal; 2 National Center for Toxicological Research, Jefferson, AR 72079, USA

**Keywords:** anti-HIV drug, nevirapine, oxidation, pyridine ring contraction

## Abstract

Nevirapine (NVP) is an anti-HIV drug associated with severe hepatotoxicity and skin rashes, which raises concerns about its chronic administration. There is increasing evidence that metabolic activation to reactive electrophiles capable of reacting with bionucleophiles is likely to be involved in the initiation of these toxic responses. Phase I NVP metabolism involves oxidation of the 4-methyl substituent and the formation of phenolic derivatives that are conceivably capable of undergoing further metabolic oxidation to electrophilic quinoid species prone to react with bionucleophiles. The covalent adducts thus formed might be at the genesis of toxic responses. As part of a program aimed at evaluating the possible contribution of quinoid derivatives of Phase I phenolic NVP metabolites to the toxic responses elicited by the parent drug, we have investigated the oxidation of 2-hydroxy-NVP with dipotassium nitroso-disulfonate (Frémy’s salt), mimicking the one-electron oxidation involved in enzyme-mediated metabolic oxidations. We report herein the isolation and full structural characterization of a 1*H*-pyrrole-2,5-dione derivative as a major product, stemming from an unusual pyridine ring contraction.

## 1. Introduction

**Figure 1:**
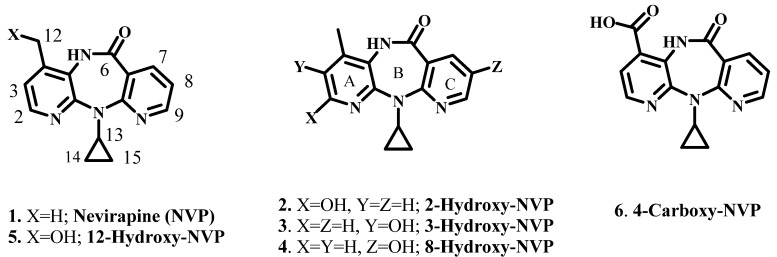
Structures of Nevirapine (**1**) and its Phase I metabolites.

Nevirapine (11-cyclopropyl-5,11-dihydro-4-methyl-6*H*-dipyrido[3,2b:2′,3′-e]diazepin-6-one (1, NVP, Figure 1) was the ﬁrst non-nucleoside reverse transcriptase inhibitor (NNRTI) approved by the US Food and Drug Administration in 1996, for use in combination therapy of HIV-1 infection. Since then, NVP has become a first-line antiretroviral agent in low resource countries, due to its availability as a generic drug and its ability to prevent vertical HIV transmission [1,2,3,4,5,6]. Despite the high efficacy of the drug, favorable lipid proﬁle [7] and suitability for use during pregnancy and breastfeeding [8,9], NVP therapy is associated with toxic events. Among these, skin rash is the most frequent and hepatotoxicity is the most severe [10]. These adverse side effects raise concerns about the chronic use of the drug, particularly in the perinatal and pediatric settings.

While the reasons for the adverse effects of NVP are still unclear, increasing evidence suggests that metabolic activation to highly reactive electrophiles, prone to react with bionucleophiles, has a role in the initiation of the toxic responses. In all species investigated, cytochrome P450 (CYP)-mediated Phase I metabolism of NVP yields 2-, 3-, 8-, and 12-hydroxy-NVP, and 4-carboxy-NVP (**2**–**6**, Figure 1) [11,12,13]. Glucuronidation, and subsequent renal excretion of the conjugates, is the major detoxification pathway for these metabolites.

**Scheme 1:**
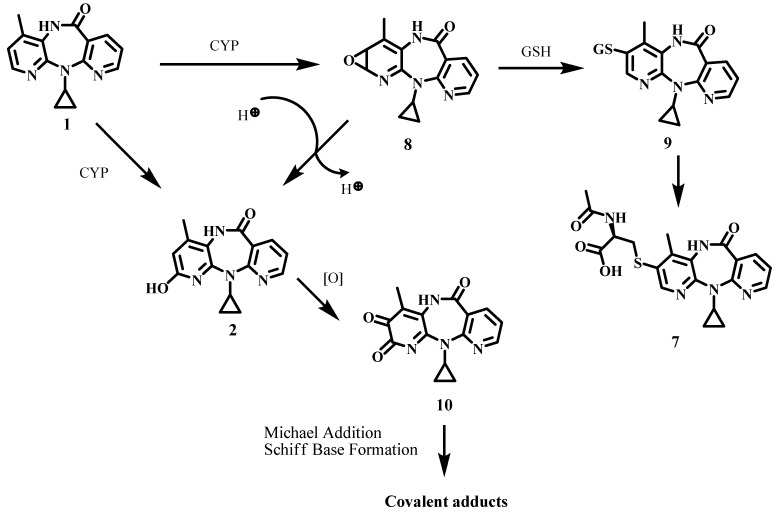
Hypothetic pathways for in vivo generation of covalent adducts from 2-hydroxy-NVP (**2**). GSH, glutathione.

It has been suggested that NVP oxidation to 12-hydroxy-NVP (**5**), probably involving subsequent Phase II activation, is the pathway responsible for a skin rash in rats that resembles the rash in humans [14,15,16]. However, other metabolic pathways may play a role in the generation of NVP-derived reactive electrophiles, as demonstrated by Srivastava *et al.* [17], who identified the mercapturate through the C3 position of NVP (**7**) in the urine of NVP-treated patients (cf. Scheme 1). This adduct was suggested to be formed by initial gluthatione (GSH) attack to an oxirane intermediate (**8**), yielding the GSH adduct **9** which underwent anabolism to **7**. Alternatively phenolic NVP metabolites may undergo metabolic activation to quinone/semiquinone electrophiles (e.g., **10**) capable of reacting with bionucleophiles through Michael-type addition and/or Schiff-base formation, leading to covalent adduct formation (Scheme 1). We have recently obtained evidence for the generation of an electrophilic quinone-imine upon chemical and enzymatic (lactoperoxidase) oxidation of the phenolic NVP metabolite, 2-hydroxy-NVP (**2**) [18]. The formation of a quinone-imine electrophile under lactoperoxidase catalysis, together with the presence of NVP in breast milk [19] and the frequent administration of the drug concurrently with breastfeeding, suggest that a quinone-imine-mediated pathway could be at the onset of adverse drug reactions in the perinatal setting. Moreover, the fact that lactoperoxidase is also abundant in tears and saliva [20] may explain NVP-induced oral and ocular toxicity [21,22].

Despite our efforts toward a thorough characterization of all reaction products, at least one product from the oxidation of **2** with dipotassium nitrosodisulfonate (Frémy’s salt), remained unidentified [18]. As a further contribution to understand the reactivity of quinoid derivatives from phenolic NVP metabolites, we report herein the isolation and full structural characterization (by NMR, MS and X-ray diffraction) of a 1*H*-pyrrole-2,5-dione derivative, stemming from an unusual pyridine ring contraction, as the major product of 2-hydroxy-NVP oxidation with Frémy’s salt.

## 2. Results and Discussion

### 2.1. Oxidation of the Metabolite 2-Hydroxy-NVP

As part of a program aimed at evaluating the possible contribution of quinoid derivatives of phenolic NVP metabolites to the toxic responses elicited by the parent drug, we investigated the oxidation of 2-hydroxy-NVP with dipotassium nitrosodisulfonate (Frémy’s salt). This reagent is frequently employed in the generation of quinones from phenolic derivatives [23,24], mimicking the one-electron oxidation involved in enzyme-mediated metabolic oxidations [25,26].

**Scheme 2:**
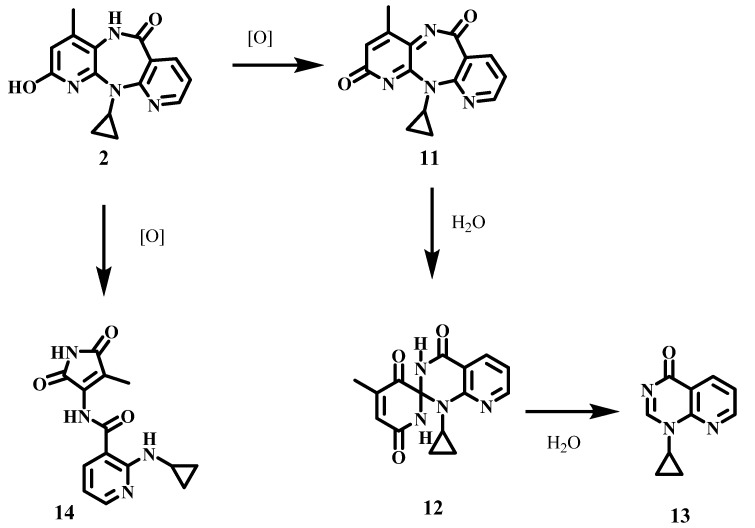
Products obtained from oxidation of 2-hydroxy-NVP with Frémy’s salt.

The reactions were conducted at room temperature using 1.3 molar equivalents of the oxidant and a biphasic system of ethyl acetate and 100 mM phosphate buffer (pH 7.4 or 10). Under these conditions, our initial studies [18] provided evidence for the rapid generation of a quinone-imine intermediate **11** (Scheme 2) that in aqueous solution underwent hydrolytic conversion to the spiro derivative **12**, which subsequently decomposed into **13**. However, the major product had not been characterized, due to ambiguities in a structural assignment based exclusively on NMR and mass spectrometry data. We have now obtained crystals suitable for X-ray diffraction, and report herein the definitive structural assignment of **14**, that was obtained in 15 and 16% yield in the oxidation of **2** with Frémy’s salt at pH 7 and 10, respectively. 

We are currently conducting experiments towards the clarification of the mechanistic pathway leading to the formation of **14**. Although still preliminary, our data indicate that **14** does not stem from quinone-imine formation. Indeed, **14** was only observed when the oxidations were conducted with Frémy’s salt, whereas the spiro derivative **12** was obtained in oxidations conducted with both Frémy’s salt and sodium periodate [18]. These observations suggest that **12** and **14** are formed via different oxidative pathways (Scheme 2).

### 2.2. Structural Characterization of the Oxidation Product *14*

**Figure 2:**
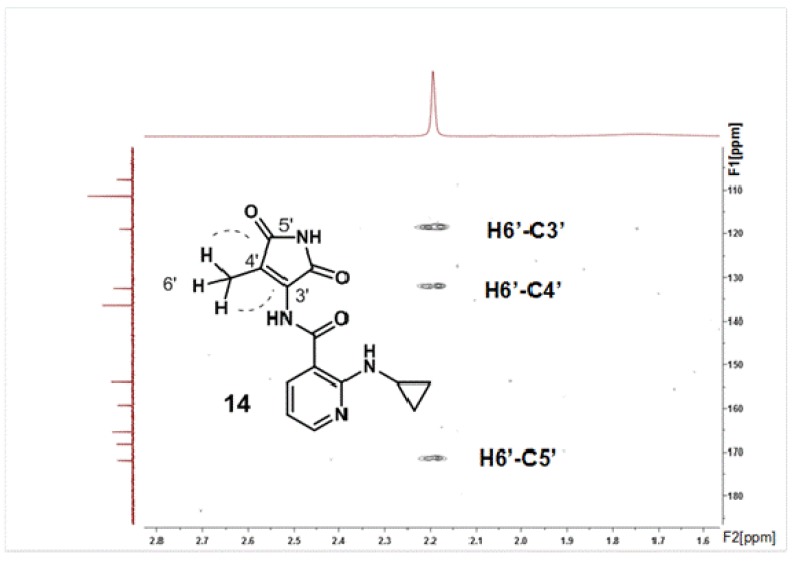
Expanded region of the ^1^H-^13^C HMBC spectrum of compound **14**, displaying the 3-bond connectivities between the methyl protons (H6′) and C5′ and C3′, and the 2-bond connectivities between the same protons and C4′.

**Figure 3:**
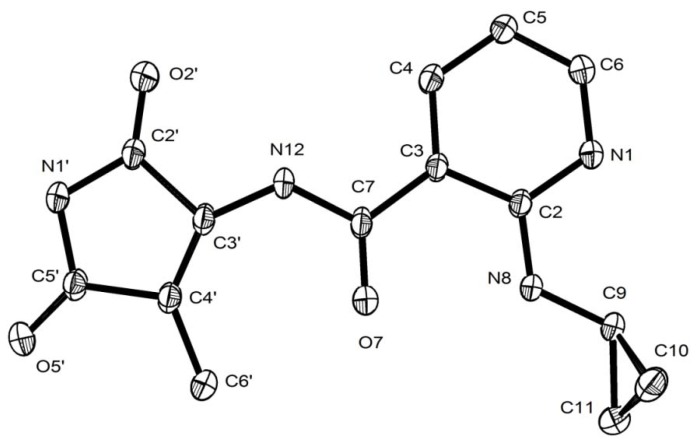
ORTEP diagram, drawn with 50% probability ellipsoids, showing the atomic labelling scheme for compound **14**.

Similarly to what was observed with all other products from 2-hydroxy-NVP oxidation [18], an initial inspection of the ^1^H- and ^13^C-NMR spectra of product **14** (cf. Experimental section) promptly allowed the conclusion that ring C (Figure 1) remained unchanged in this derivative, whereas a substantial degradation of rings A and B had occurred during the oxidation process. The lack of aromatic protons on ring A and the 3-bond ^1^H-^13^C correlation observed on the HMBC spectrum (Figure 2) between the methyl protons (H6’, 2.19 ppm) and carbon C5’ (171.8 ppm), with a resonance compatible with a carbonyl group, initially suggested the formation of a quinone structure, which was in agreement with the presence of three carbonyl groups in the molecule, inferred from the IR and ^13^C NMR data (cf. Experimental section). However, the presence of three labile protons in the ^1^H-NMR spectrum, together with the presence of only 13 distinct carbons in the ^13^C-NMR spectrum and the indication from the mass spectral data, obtained by electrospray ionization (ESI), that the protonated molecule had *m/z* 287, were not consistent with a quinone structure. Conclusive evidence for the structural assignment was only obtained by X-ray diffraction, which showed that **14** is composed of a nicotinamide framework with a cyclopropylamino substituent at position C2 and a methyl-2,5-dioxo-2,5-dihydro-1*H*-pyrrole substituent at the amide nitrogen (Figure 3), stemming from ring B opening and an unusual pyridine ring A contraction of the parent compound, 2-hydroxy-NVP.

The X-ray crystallographic data indicated that **14** crystallized in the triclinic P-1 space group, with two crystallographically independent molecules in the asymmetric unit. Both types of molecules of compound **14** include a large planar system, with bonds displaying angles close to 120° between them (see Table 1), composed by all the N atoms, as well as all C atoms except C10.

## 3. Experimental

### 3.1. Chemicals

NVP was purchased from Cipla (Mumbai, India). All other commercially available reagents and enzymes were acquired from Sigma-Aldrich Química, S.A. (Madrid, Spain), unless speciﬁed otherwise, and were used as received. 2-Hydroxy-NVP was prepared by reaction with silver acetate/iodine, followed by basic hydrolysis, as described in Antunes *et al.* [18]. Whenever necessary solvents were puriﬁed by standard methods [27].

### 3.2. General

Infrared (IR) spectra were recorded on a Perkin-Elmer 683 FTIR spectrometer; group frequencies are reported in cm^-1^. ^1^H-NMR spectra were recorded on Bruker Avance III 500 spectrometer (Bruker BioSpin GmbH, Rheinstetten, Germany) operating at 500 MHz. ^13^C-NMR spectra were recorded on the same instrument, operating at 125.77 MHz. Chemical shifts are reported in ppm downﬁeld from tetramethylsilane, and coupling constants (*J*) are reported in Hz. The presence of labile protons was conﬁrmed by chemical exchange with D_2_O. Resonance and structural assignments were based on the analysis of coupling patterns, including the ^13^C-^1^H coupling proﬁles obtained in bidimensional heteronuclear multiple bond correlation (HMBC) and heteronuclear single quantum coherence (HSQC) experiments, performed with standard pulse programs. Mass spectra were performed with a Varian system consisting of a 500-MS ion trap mass spectrometer, with an ESI ion source (Varian, Inc., Palo Alto, CA). Data acquisition and processing were performed using Varian MS Control 6.9 software. High resolution ESI mass spectra were obtained on a Bruker Apex Ultra FTICR mass spectrometer (Bruker Daltonics, Billerica, MA) at the FCUL node of the Portuguese MS network. 

**Table 1:** Bond lengths and angles for compound **14**.

**Bonds (Å)**			**Angles (°)**		
	**Molecule 1**	**Molecule 2**		**Molecule 1**	**Molecule 2**
O5’-C5’	1.2130(14)	1.2138(14)	C2’-N1’-C5’	109.18(10)	109.03(10)
O2’-C2’	1.2146(14)	1.2111(14)	N1’-C5’-C4’	108.74(10)	109.01(9)
O7-C7	1.2234(14)	1.2219(14)	C3’-C4’-C5’	105.96(9)	105.58(10)
N1’-C5’	1.3981(15)	1.3967(15)	C2’-C3’-C4’	109.12(10)	109.50(9)
N1’-C2’	1.3716(15)	1.3715(14)	N1’-C2’-C3’	106.95(9)	106.81(9)
N12-C3’	1.3789(14)	1.3814(14)	O5’-C5’-C4’	126.75(10)	126.31(11)
N12-C7	1.3750(14)	1.3735(15)	O5’-C5’-N1’	124.50(10)	124.67(11)
N1-C2	1.3553(14)	1.3497(14)	O2’-C2’-N1’	128.46(11)	128.16(11)
N1-C6	1.3378(14)	1.3380(16)	O2’-C2’-C3’	124.60(10)	125.00(10)
N8-C2	1.3482(14)	1.3498(14)	C4’-C3’-N12	138.20(10)	137.70(11)
N8-C9	1.4329(15)	1.4299(15)	N12-C3’-C2’	112.67(10)	112.79(9)
C4’-C5’	1.4956(16)	1.4967(16)	C5’- C4’-C6’	119.03(10)	118.50(10)
C3’-C4’	1.3517(15)	1.3480(15)	C3’-C4’-C6’	135.01(11)	135.88(11)
C2’-C3’	1.5094(15)	1.5087(16)	C7-N12-C3’	128.53(10)	128.24(10)
C3-C7	1.4806(16)	1.4792(15)	O7-C7-N12	120.00(11)	120.10(10)
C2-C3	1.4408(15)	1.4363(15)	N12-C7-C3	116.55(10)	116.63(9)
C5-C6	1.3836(16)	1.3817(19)	O7- C7-C3	123.44(10)	123.26(10)
C4-C5	1.3826(17)	1.3831(18)	C2-C3-C7	120.17(9)	120.31(9)
C3-C4	1.3925(15)	1.3855(16)	C4-C3-C7	122.84(10)	122.47(10)
C9-C11	1.4933(16)	1.4958(19)	C2-C3-C4	116.99(10)	117.20(10)
C9-C10	1.4975(18)	1.4966(18)	N1-C2-C3	121.63(10)	121.96(10)
C10-C11	1.5029(18)	1.4973(19)	C2-N1-C6	118.19(10)	117.84(10)
**Torsion Angles (°)**			N1-C6-C5	124.34(11)	124.44(12)
			C4-C5-C6	117.81(10)	117.79(12)
N8-C9-C11-10	−110.69(13) 70.12(12)		C3-C4-C5	120.92(10)	120.71(12)
			N8-C2-C3	121.32(10)	121.47(10)
**Angles between planes (°)**			N1-C2-N8	117.04(10)	116.57(10)
			C2-N8-C9	123.66(10)	123.40(10)
Cyclopropyl-Main frame	72.62(7)	61.55(7)	N8-C9-C11	117.54(10)	116.40(11)
			N8-C9-C10	120.07(11)	119.08(11)
			C9-C11-C10	59.97(8)	60.00(9)
			C9-C10-C11	59.70(8)	59.95(9)
			C10-C9-C11	60.33(8)	60.05(9)

X-ray crystallographic data were collected from crystals using an area detector diffractometer (Bruker AXS-KAPPA APEX II) equipped with an Oxford Cryosystem open ﬂow nitrogen cryostat at 150 K and graphite-monochromated MoKa (λ = 0.71073 Å) radiation. Cell parameters were retrieved using Bruker SMART software and reﬁned with Bruker SAINT [28] on all observed reﬂections. Absorption corrections were applied using SADABS [29]. The structures were solved by direct methods using SIR 97 [30] and reﬁned with full-matrix least-squares reﬁnement against F2 using SHELXL-97 [31]. All the programs are included in the WINGX package (version 1.70.01) [32]. All non-hydrogen atoms were reﬁned anisotropically, and the hydrogen atoms were inserted in idealized positions, riding on the parent C atom, except for the methyl hydrogens, whose orientation was reﬁned from electron density, allowing the reﬁnement of both C–C torsion angles and C–H distances, and the hydrogen atoms bonded to nitrogens, which were found directly in the density map. Drawings were made with ORTEP3 for Windows [33]. Intermolecular interactions were analysed using Mercury 1.4.2 (Build 2). Plane calculations were made using Parst [34,35]. Crystals had good quality and diffracting power, presenting low R_int_ (0.0364) values that allowed to obtain low R values (R1 all = 0.0827 and R1 obs = 0.0498) after refining. The structure was, therefore, unequivocally determined and is in good agreement with the remaining spectral characterization data for **14**. Relevant details of the X-ray data analysis are displayed in **Table 1**. Crystallographic data for **14** were deposited with the Cambridge Crystallographic Data Centre (CCDC 848449) and can be obtained free of charge from: CCDC, 12 Union Road, Cambridge CB2 1EZ, UK (Fax: +44-1223-336033; e-mail: deposit@ccdc.cam.ac.uk; http://www.ccdc.cam.ac.uk/deposit).

### 3.3. General Procedure for 2-Hydroxy-NVP (2) Oxidation

To a solution of 2-hydroxy-NVP (40 mg, 142 mmol) in ethyl acetate (8 mL) was added a solution of Frémy’s salt (48 mg, 179 mmol) in 100 mM phosphate buffer (4 mL; pH 7.4, or 10) and the mixture was stirred overnight at room temperature. Following phase separation and additional extraction with ethyl acetate, the organic layers were dried over anhydrous sodium sulfate and the products were isolated by PTLC on silica (1:1 dichloromethane/ethyl acetate). 

#### 3.3.1. At pH 7.4

*2-Cyclopropylamino-*N*-(4′-methyl-2′,5′-dioxo-2′,5′-dihydro-1*H*-pyrrol-3′-yl)pyridine-3-carboxamide* **(14)**. Obtained in 15% yield (6.2 mg). IR (KBr): 1721 (C=O), 1685 (C=O), 1660 (C=O). ^1^H-NMR (CDCl_3_): 8.46–8.45 (1H, m, H6), 8.16 (1H, bs, NH), 8.03 (1H, bs, NH), 7.80 (1H, d, *J* = 7.0, H4), 7.59 (1H, bs, NH), 6.67–6.65 (1H, m, H5), 2.92–2.93 (1H, m, H9), 2.19 (3H, s, CH_3_), 0.89–0.88 (2H, m, H10+H11), 0.59 (2H, bs, H10+H11). ^13^C-NMR (CDCl_3_): 171.8 (C5’), 168.3 (C2’/C7), 165.5 (C2’/C7), 159.3 (C2), 154.1 (C6), 136.5 (C4), 132.6 (C4’), 119.4 (C3’), 111.4 (C5), 107.8 (C3), 23.8 (C9), 10.8 (CH_3_), 7.20 (C10+C11). MS (ESI): *m/z* 287 [MH]^+^, 161 [MH-(3-amino-4-methyl-1*H*-pyrrole-2,5-dione)]^+^, 133 [161-C2H4]+, 121 [161-cyclopropyl+H]+. HRMS calcd for [C_14_H_15_N_4_O_3_]^+^, 287.11280. Found: 287.11309. For the X-ray diffraction data see the Results and Discussion section.

*1′-Cyclopropyl-4-methyl-1*H*,1′*H*-spiro[pyridine-2,2′-pyrido[2,3-d]pyrimidine]-3,4′,6(3′*H*)-trione* (12). Obtained in 7% yield. Spectroscopic data according to Antunes *et al.* [18].

#### 3.3.2. At pH 10

*2-Cyclopropylamino-N-(4′-methyl-2′,5′-dioxo-2′,5′-dihydro-1*H*-pyrrol-3′-yl)pyridine-3-carboxamide* (14). Obtained in 16% yield (6.4 mg). 

*1′-Cyclopropyl-4-methyl-1*H*,1′*H*-spiro[pyridine-2,2′-pyrido[2,3-d]pyrimidine]-3,4′,6(3′*H*)-trione* (12). Obtained in 8.3% yield (3.5 mg). Spectroscopic data according to Antunes *et al.* [18].

*1-Cyclopropylpyrido[2,3-d]pyrimidin-4(1*H*)-one* (13). Obtained in 10.5% yield (2.8 mg). Spectroscopic data according to Antunes *et al.* [18].

## 4. Conclusions

The oxidation of the phenolic NVP metabolite 2-hydroxy-NVP with Frémy’s salt, both at pH 7.4 and pH 10, yielded the 1*H*-pyrrole-2,5-dione derivative **14** as a major product, stemming from an unusual pyridine ring contraction. Although the significance of this NVP derivative *in vivo* remains to be established, the considerable structural degradation of the parent drug, leading to a mass increment inconsistent with that expected from direct oxidation alone, may explain why **14** has eluded detection in previous NVP metabolism studies, both *in vitro* and *in vivo*, which have been conducted with LC-MS detection. The availability of this fully characterized oxidation product is a valuable tool to assess its formation *in vivo*, as a further effort to establish the metabolic pathways that convert NVP into reactive electrophiles. Based upon structural considerations, reaction of **14** with bionucleophiles is conceivable, and a potential role for this compound in the onset of toxic responses elicited by NVP cannot be excluded. This new NVP derivative is now accessible for further molecular toxicology studies that are expected to clarify the relevance of phenolic NVP metabolites and their oxidation products to the toxic events associated with the parent drug.
